# Papel da Tomografia Computorizada na Exclusão de Síndrome Coronária Aguda em Contexto de Urgência: A Anatomia é o Caminho?

**DOI:** 10.36660/abc.20220273

**Published:** 2022-05-04

**Authors:** Nuno Bettencourt

**Affiliations:** 1 Universidade do Porto Faculdade de Medicina Unidade de Investigação Cardiovascular Porto Portugal Universidade do Porto Faculdade de Medicina - Unidade de Investigação Cardiovascular, Porto – Portugal

**Keywords:** Doenças Cardiovasculares, Fatores de Risco, Dor Torácica, Diagnóstico por Imagem, Angiotomografia Coronária Raios X/métodos, Sistema Único de Saúde (SUS

A dor torácica é um dos motivos mais frequentes de recurso aos serviços de urgência (SU). Apesar de apenas uma pequena minoria destes recursos corresponder a uma síndrome coronária aguda (SCA), a potencial gravidade de um diagnóstico incorreto ou de um não-diagnóstico leva a que sejam sistematicamente aplicados protocolos de confirmação ou de exclusão de doença coronária (DC) como causa da sintomatologia. Nos últimos anos o estudo seriado dos níveis sanguíneos de troponina de elevada sensibilidade (T-hs) impôs-se como um método seguro e eficaz neste contexto e é amplamente aplicado nos SU de todo mundo.^1 - 3^ No entanto, esta abordagem está associada a tempos de permanência prolongados nos SU e não é isenta de erros, sendo que alguns pacientes com clínica de angina instável podem não ser detectados pelo recurso a marcadores de necrose miocárdica, mesmo que de elevada sensibilidade.

É neste contexto que novas abordagens de exclusão de DC com recurso a métodos de imagem ganham relevância e têm gerado um grande interesse por parte da comunidade científica. Pelo seu elevado desempenho diagnóstico, a AngioTC cardíaca (ATC) assume-se como o principal candidato a alterar o *status-quo* da seriação enzimática nestas situações. Trata-se de um exame simples, rápido, robusto e com um elevado valor preditivo negativo – o que a torna particularmente adequada em contextos de SU em que é essencial excluir de forma rápida e eficaz a presença de DC. Os estudos ROMICAT foram dos primeiros a documentar as vantagens da ATC neste contexto e a salientar o seu valor prognóstico aditivo em relação ao score de risco TIMI (TIMI Sc). Porém, tal como a grande maioria dos estudos que utilizaram ATC em contexto de SU hospitalar, aplica-se maioritariamente a populações de baixo risco.^4 , 5^

Neste número da revista, Matos Soeiro et al.,^6^ publicam um interessante trabalho que compara o desempenho da ATC com a medição seriada de T-hs em 100 pacientes que recorreram ao SU por dor torácica, com ECG e troponina inicial negativos e risco intermédio de eventos segundo o TIMI Sc.^6^ De forma pouco surpreendente, a ATC apresentou melhor desempenho do que as medidas de T-hs na detecção de DC importante, sendo que o intervalo de tempo entre a chegada do paciente e a ATC foi aproximadamente uma hora menor que o intervalo entre a chegada do paciente e o resultado da segunda medida de troponina. O estudo tentou também fazer uma avaliação do impacto clínico em termos de hospitalização, morte, e infarto agudo do miocárdio aos 30 dias, através de um seguimento telefónico, mas o pequeno tamanho amostral e a reduzida taxa de eventos (2%) impossibilitaram a detecção de eventuais diferenças entre as duas abordagens. Ainda assim, com base nestes resultados, os autores sugerem que pacientes em risco intermediário sem alterações isquêmicas no eletrocardiograma deveriam ser preferencialmente estratificados na admissão por ATC uma vez que esta é mais eficiente na identificação de DC.

Este estudo acrescenta alguma informação adicional ao conhecimento prévio uma vez que se aplica a populações de risco intermédio, mas os seus resultados devem ser interpretados com cuidado. Muito mais do que documentar a baixa concordância entre as medidas de troponina e a presença de lesões coronárias significativas interessa-nos, sobretudo documentar qual a estratégia mais custo-eficaz na exclusão de uma SCA em contexto de recurso ao SU por clínica de dor torácica – algo que não é muitas vezes avaliável com recurso apenas a testes anatómicos como a ATC. Tal como referimos a probabilidade de ocorrerem falsos negativos com o uso da estratégia baseada na seriação enzimática, também a documentação de DC anatomicamente relevante (definida pela presença de estenose coronária ≥50%), não assegura que ela seja responsável pela sintomatologia e que estejamos perante uma SCA, uma vez que em populações de risco intermédio como a estudada, a presença de DC estável e assintomática não é rara e pode ser apenas um “ *bystander* ” inocente, sem qualquer impacto funcional no caso em estudo. Deste modo, à luz da evidência atual, devemos ser prudentes na adoção sistemática desta estratégia no sentido de evitar sobrediagnósticos e “sobretratamento” com revascularizações inadequadas – que têm vindo a demostrar efeitos deletérios no contexto de doença coronária estável. Com base neste estudo unicêntrico, com um tamanho amostral pequeno e um número de eventos clínicos muito reduzido, não é possível afirmar a eficácia e adequação de testar sistematicamente com ATC pacientes que recorrem ao SU por clínica de dor torácica e risco intermédio de eventos. Ainda assim, este trabalho é mais uma contribuição importante para salientar o papel que a ATC pode ter na orientação clínica destes pacientes e junta-se à longa evidência que documenta as mais-valias desta técnica em diferentes cenários e à imperiosa necessidade de disponibilizar esta tecnologia nos serviços nacionais de saúde do século XXI.^7 - 10^
