# Supplementary material for: Papel da Tomografia Computorizada na Exclusão de Síndrome Coronária Aguda em Contexto de Urgência: A Anatomia é o Caminho?
Source: Arq Bras Cardiol. 2022 May 4;118(5):903–4. [Article in Portuguese] doi: 10.36660/abc.20220273 (PMC9368882; doi:10.36660/abc.20220273)
Supplement: Translation

# Translation: Role of Computed Tomography in Excluding Acute Coronary Syndrome: is Anatomy the Way?

**Authors:** Nuno Bettencourt

**Affiliations:** 1 Portugal Universidade do Porto Faculdade de Medicina - Unidade de Investigação Cardiovascular, Porto – Portugal

**Keywords:** Cardiovascular Diseases, Risk Factors, Chest Pain, Diagnostic.Imaging, Tomography,X-Ray Computed/methods

Chest pain is one of the most common reasons for admission to the emergency room (ER). Although only a small minority of these correspond to an acute coronary syndrome (ACS), the potential severity of a misdiagnosis or a non-diagnosis implies using systematic protocols to confirm or exclude coronary artery disease (CAD) as a cause of symptoms. In recent years, the sequential blood testing of high-sensitive troponin (cTnI-hs) levels has become a safe and effective method in this context and is commonly applied worldwide.^1 - 3^ However, this approach is associated with prolonged times in the ER and is not error-free, as some patients with unstable angina may not be correctly identified.

New approaches using imaging methods to exclude CAD gained relevance and have generated interest from the scientific community. Cardiac CT angiography (CTA), due to its high diagnostic performance, is assumed to be the main candidate to change the *status-quo* of the current approach based on serial blood testing. It is a simple, fast and robust test with a high negative predictive value – which makes it particularly suitable in ER, where it is essential to quickly and effectively exclude the presence of CAD. The ROMICAT studies were among the first to document the advantages of CTA in this context and to emphasize its additive prognostic value concerning the TIMI risk score (TIMI RS). However, like the vast majority of studies that used CTA in the context of hospital ER, their results mostly apply to low-risk populations.^4 , 5^

In this issue of the journal, Matos Soeiro et al.^6^ publish an interesting study that compares the performance of CTA against the serial assessment of cTnI-hs in 100 patients with chest pain referred to the ER, initially negatives ECG and cTnI-hs and an intermediate TIMI RS.^6^ Unsurprisingly, CTA performed better than cTnI-hs measurements in detecting important CAD, with the time interval between patient arrival and CTA being approximately one hour shorter than the interval between patient arrival and the result of the second troponin measurement. The study also attempted to assess the clinical impact of hospitalization, death, and acute myocardial infarction at 30 days through telephone follow-up. However, the small sample size and low event rate (2%) made detecting any potential differences between the two approaches impossible. Even so, based on these results, the authors suggest that patients at intermediate risk without ischemic changes on the electrocardiogram should preferably be stratified at admission by CTA, as it is more efficient in identifying CAD.

This study adds information to previous scientific knowledge as it applies to intermediate-risk populations, but its results should be interpreted cautiously. More important than documenting the low agreement between troponin measurements and the presence of coronary lesions is finding the most cost-effective strategy for excluding an ACS in the context of ER admissions due to chest pain – which is sometimes impossible using only anatomical tests such as CTA. Just as we mentioned the probability of false negatives with the use of a strategy based on serial blood-testing, the documentation of anatomically relevant CAD (defined by the presence of coronary stenosis ≥50%) does not guarantee that it is responsible for the symptoms and that we are facing an ACS. In intermediate-risk populations such as the one studied, the presence of stable and asymptomatic CAD is not rare, and it may be an innocent bystander without any functional and clinical impact. Therefore, in light of the current evidence, we must be cautious in systematically adopting this strategy to avoid overdiagnosis and “overtreatment” with inadequate revascularizations – which have been proven to have deleterious effects in the context of stable coronary disease. Based on this single-center study, with a small sample size and a very small number of clinical events, it is impossible to affirm the efficacy and adequacy of systematically testing with CTA all the patients coming to the ER due to chest pain and an intermediate risk of events. This work is yet another important contribution to highlighting the role that CTA can play in the management of these patients. It adds to the long evidence that documents the benefits of this technique in different scenarios and the imperative need to make this technology available in the national health services of the 21^st^century.^7 - 10^
